# Role of the Water–Metal Ion Bridge in Quinolone Interactions with *Escherichia coli* Gyrase

**DOI:** 10.3390/ijms24032879

**Published:** 2023-02-02

**Authors:** Hannah E. Carter, Baylee Wildman, Heidi A. Schwanz, Robert J. Kerns, Katie J. Aldred

**Affiliations:** 1 Biology Department, University of Evansville, Evansville, IN 47722, USA; 2 Department of Pharmaceutical Sciences and Experimental Therapeutics, University of Iowa, Iowa City, IA 42232, USA

**Keywords:** quinolone, quinolone resistance, water–metal ion bridge, gyrase, topoisomerase

## Abstract

Fluoroquinolones are an important class of antibacterials, and rising levels of resistance threaten their clinical efficacy. Gaining a more full understanding of their mechanism of action against their target enzymes—the bacterial type II topoisomerases gyrase and topoisomerase IV—may allow us to rationally design quinolone-based drugs that overcome resistance. As a step toward this goal, we investigated whether the water–metal ion bridge that has been found to mediate the major point of interaction between *Escherichia coli* topoisomerase IV and *Bacillus anthracis* topoisomerase IV and gyrase, as well as *Mycobacterium tuberculosis* gyrase, exists in *E. coli* gyrase. This is the first investigation of the water–metal ion bridge and its function in a Gram-negative gyrase. Evidence suggests that the water–metal ion bridge does exist in quinolone interactions with this enzyme and, unlike the Gram-positive *B. anthracis* gyrase, does use both conserved residues (serine and acidic) as bridge anchors. Furthermore, this interaction appears to play a positioning role. These findings raise the possibility that the water–metal ion bridge is a universal point of interaction between quinolones and type II topoisomerases and that it functions primarily as a binding contact in Gram-positive species and primarily as a positioning interaction in Gram-negative species. Future studies will explore this possibility.

## 1. Introduction

*Escherichia coli* is a Gram-negative bacillus that is a common pathogen, as well as a model organism. Treatment for ailments caused by *E. coli* often includes a fluoroquinolone antibacterial [1]. The most commonly prescribed fluoroquinolones are ciprofloxacin (“Cipro”) and levofloxacin (“Levaquin”) [2,3]. Like many antibacterial agents, the clinical efficacy of the fluoroquinolone class is threatened by increasing rates of resistance [4,5,6,7,8,9].

Fluoroquinolones kill bacteria by poisoning the activity of type II topoisomerases—essential enzymes that regulate DNA topology [5,8,9,10,11]. Most bacterial species, including *E. coli*, contain two type II topoisomerases, gyrase and topoisomerase IV. Gyrase primarily functions ahead of the replication fork to relieve supercoiling that arises due to unwinding of the DNA helix. Meanwhile, topoisomerase IV works primarily behind the fork to resolve knots and tangles that arise in the genome. Topoisomerase IV also functions as an efficient decatenase to separate daughter chromosomes following replication [4,12,13,14,15,16].

Both type II topoisomerases must introduce double-strand DNA breaks into the genome in order to carry out their essential functions [12,13,16]. Briefly, the enzymes cut both strands of the “gate-segment” of DNA and covalently attach to the newly generated termini, creating a structure known as a “cleavage complex”. Then, they pass a “transfer-segment” of DNA through the break. This is followed by religation of the break and release of both DNA helices. Fluoroquinolones take advantage of this double-strand break mechanism and insert into the cut to prevent the enzymes from repairing the damage they created, thereby increasing the number of cleavage complexes in the cell. As a result of this blockage and build-up of cleavage complexes, double-strand breaks accumulate in the cell and overwhelm repair systems, ultimately leading to cell death [2,8,9,17,18,19,20].

**Figure 1:**
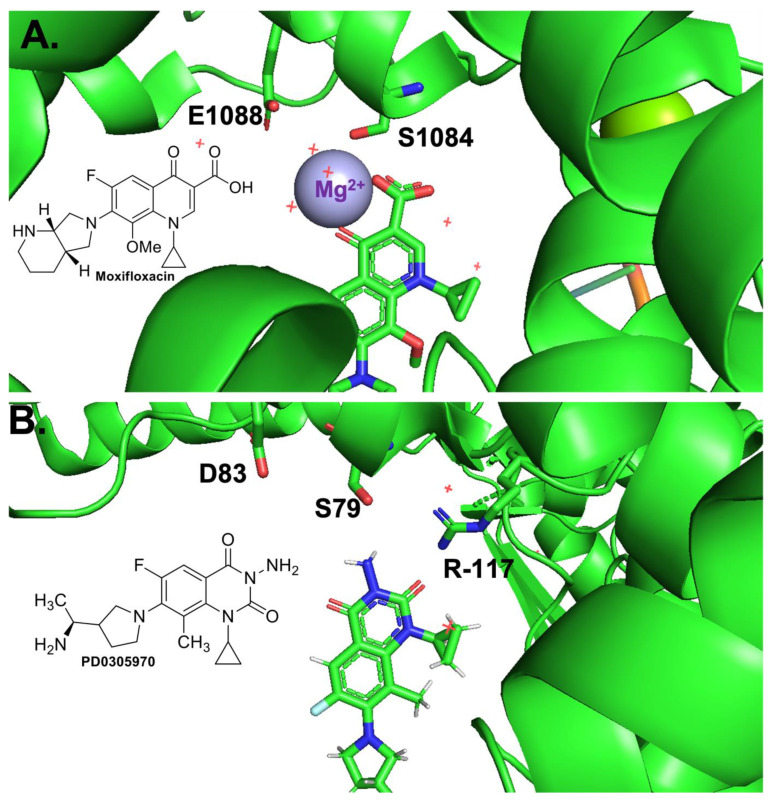
Crystal structures of a quinolone and a quinazolinedione interacting with topoisomerase IV-DNA cleavage complexes. (**A**) Crystal structure of moxifloxacin, DNA, and *A. baumannii* topoisomerase IV. Fluoroquinolone and key residues displayed as sticks, water molecules displayed as red +, divalent magnesium ion displayed in lavender. Adapted from RCSB PDB: 2XKK, visualized with The PyMOL Molecular Graphics System, Version 2.5.2 Schrödinger, LLC. Chemical structure of Moxifloxacin is shown in the inset. (**B**) Crystal structure of PD0305970, DNA, and *S. pneumoniae* topoisomerase IV. Quinazoline-2,4-dione displayed as sticks. Adapted from RCSB PDB: 3RAF, visualized with The PyMOL Molecular Graphics System, Version 2.5.2 Schrödinger, LLC. Chemical structure of PD0305970 is shown in the inset.

Typically, specific mutations must occur in both gyrase and topoisomerase IV in order to generate clinically relevant levels of resistance. The most common mutations in both enzymes across a range of species have been found to be at the positions equivalent to Ser83 and Asp87 in *E. coli* gyrase [1,4,7,10,11,21,22,23,24,25,26,27]. In *E. coli* gyrase, the specific mutations most commonly observed are S83L and D87N or D87Y [28]. Previous biochemical work with *E. coli* topoisomerase IV [29], *Bacillus anthracis* topoisomerase IV [28,30], *Mycobacterium tuberculosis* gyrase [31], and *B. anthracis* gyrase [32] have indicated that these residues coordinate a water–metal ion bridge interaction (first suggested by an x-ray crystallography structure of a cleavage complex formed between *Acinetobacter baumannii* topoisomerase IV and moxifloxacin [33]) that serves as the main interaction point between the drug and enzyme. In the water–metal ion bridge (Figure 1), the C3/C4 keto acid of the quinolone coordinates a Mg^2+^ ion. The hydration sphere of this ion is filled out by four water molecules, two of which are coordinated to the enzyme through hydrogen bonds to Ser83 and Asp87 [28,33]. Interestingly, this bridge has been found to provide different functions in different enzymes. In *B. anthracis* topoisomerase IV [28,30] and gyrase [32], it functions primarily as a binding contact, while in *E. coli* topoisomerase IV [29] it functions primarily as a positioning contact. Based on these limited examples, it appears that the water–metal ion bridge coordinated by “Ser83” and “Asp87” could be universal and that it could serve different functions based on the Gram classification of the bacterial species in question.

**Figure 2:**
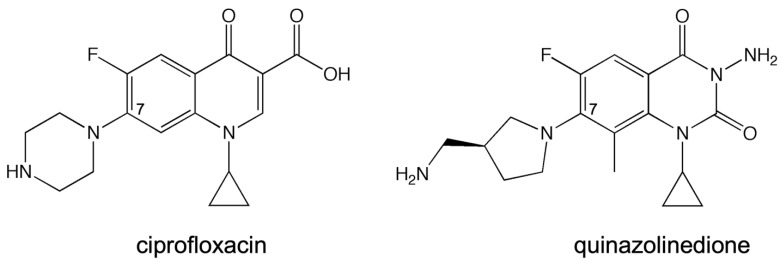
Structures of the quinolone (ciprofloxacin) and quinazolinedione used in this study. The C-7 position is labeled.

In the aforementioned biochemical studies that tested for the presence and function of the water–metal ion bridge interaction [28,29,30,31,32], a quinazolinedione (which lacks the C3/C4 keto acid found in quinolones) has been used as the comparison drug due to its apparent metal-ion-independent function and its ability to overcome resistance caused by mutations at the conserved serine and acidic residues [34]. Based on a crystal structure of a cleavage complex formed by *Streptococcus pneumoniae* topoisomerase IV in the presence of the quinazolinedione PD0305970, quinazolinediones do not interact with the enzyme through either conserved residue, nor do they use a metal ion in any manner. Thus, they serve as a valuable comparator when examining the effects of metal ion variation on drug activity against the target enzymes and when examining the effects of enzyme mutations on drug activity. In this study, the quinolone that was used was ciprofloxacin (Figure 2), which differs from moxifloxacin (used in the crystallographic study; see Figure 1A, inset) only at the C-7 position. In addition, the quinazolinedione that was used here (Figure 2) differs from PD0305970 (used in the crystallographic study; see Figure 1B, inset) only in the absence vs. presence of a methyl group on the C-7 substituent. In both cases, these substituents are not predicted to play a role in drug–enzyme interaction based on the crystallographic studies, nor are they implicated to be involved based on the biochemical data from other topoisomerases in which the water–metal ion bridge has been investigated. 

Here, the universality of the water–metal ion bridge and its possible split of function as a binding vs. positioning contact in Gram-positive vs. Gram-negative species will be further explored. Specifically, *E. coli* gyrase was examined, as there is yet to be a published example of a Gram-negative gyrase that addresses the existence and function of the water–metal ion bridge. We found that the water–metal ion bridge likely does exist as the major point of interaction between quinolones and *E. coli* gyrase, is likely anchored by the conserved serine and acidic residues, and appears to function as a positioning contact. Future studies aim to explore additional common pathogenic species of both Gram-positive and Gram-negative bacteria to further address the seeming universality of the bridge interaction and its apparent split of function down Gram-positive and Gram-negative lines.

## 2. Results and Discussion

**Figure 3:**
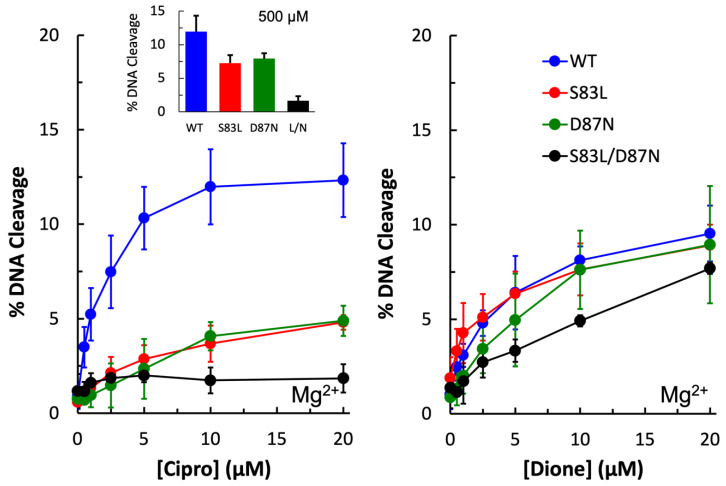
Plasmid DNA cleavage induced by wild-type (blue), GyrA^S83L^ (red), GyrA^D87N^ (green), and GyrA^S83L/D87N^ (black) gyrase enzymes in the presence of 0–20 µM ciprofloxacin (**left**) or quinazolinedione (**right**). Mg^2+^ was the metal ion. The inset in the left panel shows the level of cleavage induced by the enzymes in the presence of 500 µM ciprofloxacin. Error bars represent the standard deviation of three or more independent experiments.

The first step toward determining whether the water–metal ion bridge exists in quinolone interactions with *E. coli* gyrase was to confirm that the purified wild-type enzyme was sensitive to ciprofloxacin and that the mutant enzymes were resistant. As seen in Figure 3 (left panel), wild-type shows increasing levels of DNA cleavage in the presence of increasing concentrations of ciprofloxacin, with maximum cleavage reached at 10 µM. In contrast, the single mutant enzymes GyrA^S83L^ and GyrA^D87N^ show significantly reduced sensitivity to the quinolone, and even at 500 µM ciprofloxacin (left panel, inset) only about 50% of the wild-type level of cleavage is reached. The double mutant gyrase GyrA^S83L/D87N^ shows essentially no sensitivity to ciprofloxacin, even at 500 µM. These findings suggest that these mutations do not cause resistance simply by decreasing binding between the drug and enzyme as high levels of the drug cannot overcome the mutations to reach wild-type levels. 

3-amino-7-[(3S)-3-(aminomethyl)-1-pyrrolidin-yl]-1-cyclopropyl-6-fluoro-8-methyl-2,4-(1H,3H)-quinazoline-dione (“quinazolinedione”) has previously been shown to be a metal-ion-independent drug and overcome quinolone resistance caused mutations at these conserved residues in a number of type II topoisomerases [28,29,30,31,32,34]. As expected, wild-type as well as all three mutant enzymes were sensitive to the quinazolinedione and showed increasing levels of cleavage in the presence of increasing drug concentrations (Figure 3, right panel).

**Figure 4:**
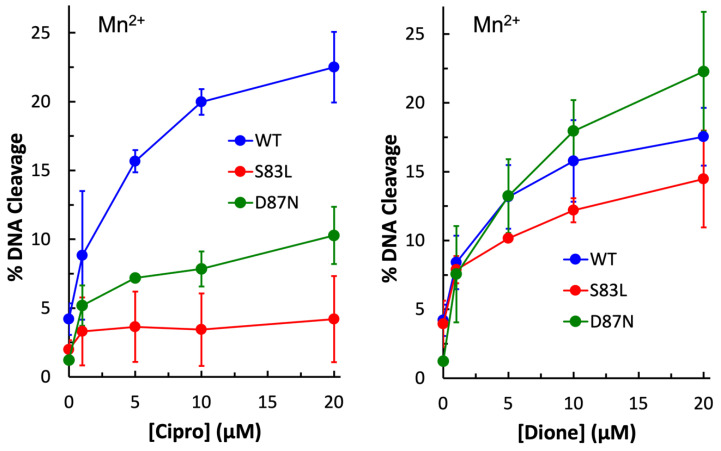
Plasmid DNA cleavage induced by wild-type (blue), GyrA^S83L^ (red), and GyrA^D87N^ (green) gyrase enzymes in the presence of 0–20 µM ciprofloxacin (**left**) or quinazolinedione (**right**). Mn^2+^ was the metal ion. Error bars represent the standard deviation of three or more independent experiments.

Next, Mn^2+^ was substituted for Mg^2+^ in cleavage reactions with ciprofloxacin and quinazolinedione with wild-type and single mutant enzymes to determine whether a metal ion plays a role in the function of the quinolone against this Gram-negative gyrase enzyme. Under these conditions, both WT and mutant enzymes induced higher levels of quinazolinedione-induced DNA cleavage than they did in the presence of Mg^2+^ (Figure 4, right panel). This was also seen with wild-type in the presence of ciprofloxacin (Figure 4, left panel). However, with the GyrA^S83L^ and GyrA^D87N^ mutant enzymes, this was not the case. With GyrA^D87N^ gyrase, ciprofloxacin-induced cleavage was approximately equal regardless of which metal ion was used. With GyrA^S83L^ gyrase, little to no increase in cleavage was observed in the presence of ciprofloxacin when Mn^2+^ was the metal ion. These findings suggest that a metal ion is indeed involved in the interaction between quinolones and the enzyme and that these residues play a role in the interaction. 

**Figure 5:**
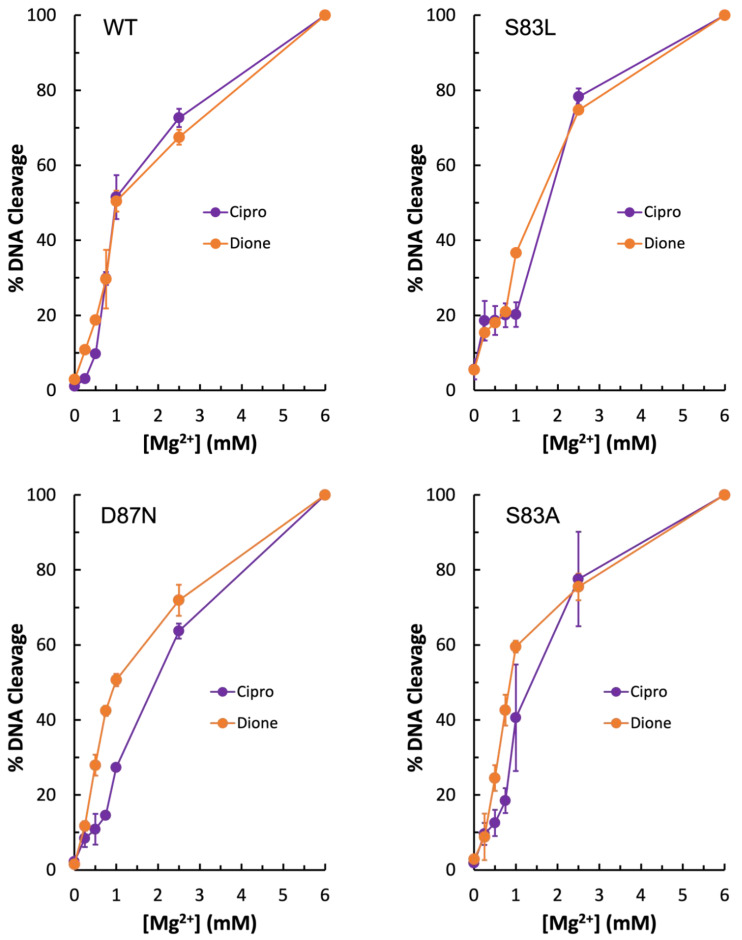
Levels of Mg^2+^ required for wild-type and mutant gyrase enzymes to induce plasmid DNA cleavage in the presence of 50 µM ciprofloxacin (purple) or quinazolinedione (orange). Wild-type is shown in the top left, GyrA^S83L^ in the top right, GyrA^D87N^ in the bottom left, and GyrA^S83A^ in the bottom right. Error bars represent the standard deviation of either two (WT, S83L, and D87N) or three (S83A) independent experiments.

To further investigate the metal ion requirement for quinolone vs. quinazolinedione activity against gyrase, Mg^2+^ ion titrations were conducted with wild-type, GyrA^S83L^, and GyrA^D87N^ gyrase enzymes. As seen in Figure 5 (top left panel), the wild-type enzyme required equivalent amounts of Mg^2+^ regardless of the drug used. However, with GyrA^D87N^ (bottom left panel), there is a right shift of the curve in the presence of the quinolone, indicating that quinolone activity requires higher concentrations of the ion. When coupled with the data shown in Figure 3 and Figure 4, this supports the conclusion that the water–metal ion bridge is the major point of interaction between quinolones and *E. coli* gyrase. Interestingly, there is little difference in Mg^2+^ requirement for ciprofloxacin and quinazolinedione with the GyrA^S83L^ mutant at most concentrations tested (Figure 5, top right panel). However, the quinolone does show a sort of plateau in the induced cleavage level at low levels of Mg^2+^ before rapidly increasing between 1 and 2 mM. This difference in shape between the quinolone and quinazolinedione curves is consistent with the metal ion playing a role in quinolone interactions with the enzyme and the quinazolinedione being a metal-ion-independent drug.

**Figure 6:**
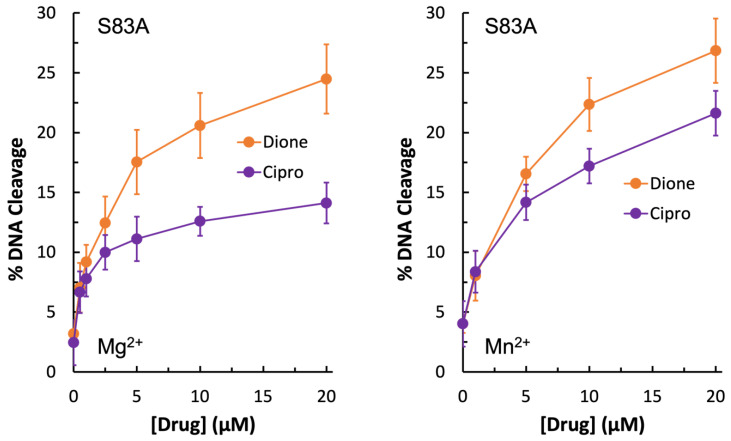
Plasmid DNA cleavage induced by GyrA^S83A^ gyrase in the presence of 0–20 µM ciprofloxacin (purple) or quinazolinedione (orange) with 6 mM Mg^2+^ (**left**) or 5 mM Mn^2+^ (**right**) as the metal ion. Error bars represent the standard deviation of three or more independent experiments.

Due to the unexpected shape of the GyrA^S83L^ Mg^2+^ titration curves, it was concluded that if the water–metal ion bridge exists in *E. coli* gyrase–quinolone interactions then the bulky leucine residue in place of the more compact serine could disrupt the quinolone-enzyme interaction beyond simply preventing coordination of the water molecules that are part of the water–metal ion bridge. For this reason, GyrA^S83A^ gyrase was generated. Unexpectedly, this mutant enzyme did not display resistance to ciprofloxacin in the presence of either Mg^2+^ or Mn^2+^ (Figure 6, left panel and right panel, respectively). In fact, much like the wild-type enzyme, GyrA^S83A^ gyrase showed increased levels of ciprofloxacin-induced DNA cleavage with Mn^2+^ as compared to Mg^2+^ (compare Figure 4, Figure 5 and Figure 6). However, cleavage induced by the quinazolinedione was nearly identical with this mutant regardless of which divalent ion was present. Because changing the metal ion affects quinolone-induced but not quinazolinedione-induced DNA cleavage, this is another piece of evidence supporting the conclusion that a metal ion is important for the interaction between quinolones and *E. coli* gyrase. Moreover, despite not causing quinolone-resistance, the GyrA^S83A^ mutant enzyme required an increased concentration of Mg^2+^ to reach maximal DNA cleavage with the quinolone as compared to the quinazolinedione (Figure 5, bottom right panel). This finding is also consistent with the water–metal ion bridge existing and Ser83 playing a role in coordinating it. Thus, it appears that the water–metal ion bridge exists in *E. coli* gyrase–quinolone interactions and that Ser83 and Asp87 act as the anchors.

**Figure 7:**
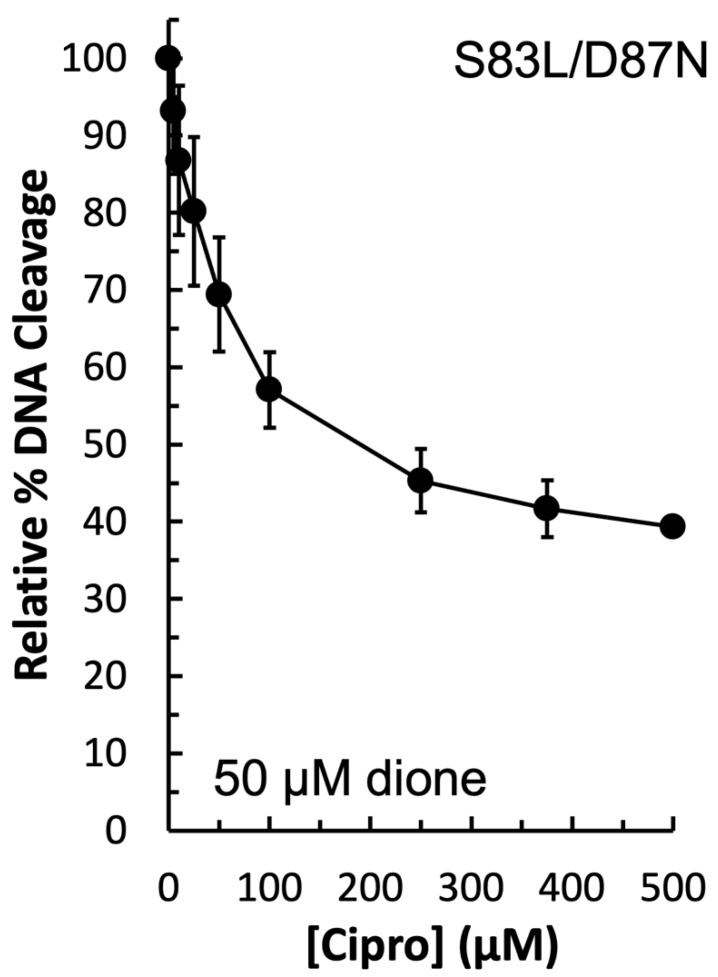
Competition between ciprofloxacin and the quinazolinedione to induce plasmid DNA cleavage by GyrA^S83L/D87N^ gyrase. An amount of 50 µM quinazolinedione (the concentration at which maximal cleavage was achieved with this drug–enzyme combination) was included simultaneously in all reactions with 0–500 µM ciprofloxacin to determine the ability of the quinolone to compete with the quinazolinedione for interaction with the enzyme. The level of cleavage seen in the presence of the quinazolinedione alone was set to 100%. Error bars represent the standard deviation of three independent experiments.

In order to determine the function of the apparent water–metal ion bridge in the interaction between *E. coli* gyrase and ciprofloxacin, a competition assay was carried out. In this assay, 50 µM quinazolinedione was used to induce cleavage by the double mutant GyrA^S83L/D87N^ enzyme in the presence of increasing concentrations of ciprofloxacin. Because ciprofloxacin did not induce DNA cleavage by this mutant enzyme (see Figure 3, left panel), then any decrease in DNA cleavage would be due to the quinolone competing out the quinazolinedione. As shown in Figure 7, ciprofloxacin was effective at competing out the quinazolinedione and decreasing DNA cleavage. Thus, it appears that the water–metal ion bridge functions primarily as a positioning interaction as the decrease in cleavage with equal concentrations of the two drugs indicates that they do not differ greatly in their ability to bind to the cleavage complex. This conclusion is consistent with the inability of high concentrations of ciprofloxacin to induce high levels of cleavage when the conserved residues are mutated (see Figure 3, left panel). Furthermore, this conclusion is consistent with the data gathered with the GyrA^S83A^ and GyrA^S83L^ mutant enzymes: it appears that the smaller alanine residue does not disrupt the bridge that is coordinated and stabilized by the remaining aspartic acid residue, while the bulkier leucine causes steric hindrance and disrupts this interaction.

## 3. Materials and Methods

### 3.1. Enzymes and Materials

Wild-type GyrA and GyrB genes from *E. coli* were PCR amplified and cloned into pET16b (Novagen, Madison, WI, USA), which added an N-terminal 10x His-tag. GyrA mutants—S83L, D87N, S83F/D87N, and S83A—were generated using a QuikChange Lightning Site-Directed Mutagenesis Kit (Agilent, Santa Clara, CA, USA). All clones, both wild-type and mutant, were sequenced prior to expression to confirm accuracy. Following expression in Rosetta 2 (DE3) pLysS *E. coli* (Novagen), cells were pelleted, resuspended in 20 mM TRIS-HCl (pH 7.9), 10% glycerol, 10 mM imidazole, 500 mM NaCl, and protease inhibitors (cOmplete, EDTA-free protease inhibitor cocktail; Roche), and lysed via sonication on ice. The lysate was cleared by centrifugation before being loaded onto a HisTrap HP column (Ge Healthcare, Chicago, IL, USA). The column was first washed with cold wash buffer (20 mM TRIS-HCl pH 7.9, 30 mM imidazole, 1 M NaCl, and protease inhibitors), then cold wash buffer containing 500 mM NaCl, and finally cold wash buffer containing 500 mM NaCl and 60 mM imidazole. Subunits were then eluted in 20 mM TRIS-HCl pH 7.9, 500 mM imidazole, 500 mM NaCl, and protease inhibitors. Finally, they were buffer exchanged into 50 mM TRIS-HCl pH 7.5, 200 mM NaCl, and 20% glycerol using Amicon Ultra-15 centrifugal filters (30K; Millipore, Burlington, MA, USA). Purity was checked via Coomassie staining, and quantification was via A_260_ reading on a NanoDrop 1000 (Thermo, Waltham, MA, USA). The resulting proteins were stored at –80 °C and used as a 1:2 GyrA:GyrB mixture in all assays. Dilution immediately prior to use was into 50 mM TRIS-HCl pH 7.9, 5 mM DTT, 30% glycerol, 125 mM potassium glutamate, and 200 mM KCl.

The negatively supercoiled pBR322 DNA substrate was purified using a Plasmid Mega Kit (Qiagen, Hilden, Germany) as described by the manufacturer. 

Ciprofloxacin (SigmaAldrich, St. Louis, MO, USA) was kept at 4 °C as a 40 mM stock solution dissolved in 0.1 N NaOH. It was diluted 5x into 10 mM TRIS-HCl pH 7.9 immediately prior to use. The quinazolinedione 3-amino-7-[(3S)-3-(aminomethyl)-1-pyrrolidin-yl]-1-cyclopropyl-6-fluoro-8-methyl-2,4-(1H,3H)-quinazoline-dione was synthesized as previously described [35]. For simplicity, it is referred to simply as “quinazolinedione” throughout this paper. It was kept at 4 °C as a 20 mM stock solution dissolved in 100% DMSO.

### 3.2. Cleavage Assays

Cleavage assays were carried out as previously described [36]. Drug titrations were conducted to test for resistance. In these assays, 100 nM gyrase was combined with 0.6 µg pBR322 and various amounts of drug (as indicated in the figures) in cleavage buffer (10 mM TRIS-HCl pH 7.9, 20 mM NaCl, 5% glycerol, 6 mM MgCl_2_, 1 mM DTT, and 1 mM ATP). For cleavage assays carried out in the presence of Mn^2+^, MnCl_2_ was substituted for MgCl_2_ in the cleavage buffer at a concentration of 5 mM. For Mg^2+^ titrations, the drug was added to the cleavage buffer while the metal ion was omitted to allow titration of MgCl_2_ from 0–6 mM as indicated in the figures. For competition assays, reactions contained a constant amount of quinazolinedione and 0–500 µM ciprofloxacin, as indicated in the figures. In all cases, the resulting 20 µL reaction was incubated at 37 °C for 10 min. Reactions were terminated and cleavage complexes were trapped by addition of 2 µL of 5% SDS. After addition of 1 µL of 250 mM Na_2_EDTA (pH 8.0) and 2 µL of 0.8 mg/mL proteinase K, samples were incubated at 45 °C for 45 min. Then, 2 µL of agarose gel loading buffer was added to each reaction, which was incubated at 45 °C for 5 min before electrophoresis on a 1% agarose gel made in 40 mM Tris-acetate (pH 8.3) and 2 mM Na_2_EDTA containing 0.5 µg/mL ethidium bromide. DNA bands were visualized using the FOTO/Analyst Luminary FX system (Fotodyne) and quantified using the stand-alone AlphaEase program (Alpha Innotech).

## 4. Conclusions

When the data presented above are viewed holistically, it is likely that the water–metal ion bridge does exist in the quinolone-*E. coli* gyrase interaction and that the conserved Ser and acidic residue 4 positions downstream (on helix 4 of the GyrA subunit) act as the bridge anchors. As seen above, metal ion substitution affected quinolone-induced but not quinazolinedione-induced DNA cleavage mediated by the target enzyme implicating a role for the metal ion in the quinolone interaction. In addition, because the mutant enzymes required higher concentrations of metal ions to reach maximal DNA cleavage in the presence of the quinolone but not the quinazolinedione, it appears that the conserved residues anchor the bridge. All of these data and conclusions are consistent with both crystal structures presented in Figure 1 in which the quinolone interacts with the enzyme via a water–metal ion bridge that is anchored by the conserved Ser and acidic residues and that the quinazolinedione is metal-ion-independent and does not utilize these anchor residues to interact with the enzyme. These findings are also consistent with the biochemical data gathered with *E. coli* topoisomerase IV [29], *B. anthracis* topoisomerase IV [28,30], and *M. tuberculosis* gyrase [31] (which is unusual because it is the only type II topoisomerase in that species and has efficient decatenase activity). However, it is in contrast to *B. anthracis* gyrase, which is a “typical” gyrase that occurs alongside topoisomerase IV and has inefficient decatenase activity, where the water–metal ion bridge appears to be anchored solely by the Ser residue [32]. Studies on the water–metal ion bridge in other “typical” gyrase enzymes, as well as topoisomerase IV enzymes, will reveal whether the bridge anchors in most gyrases are consistent with those in topoisomerase IV or whether “typical” gyrases most often rely on only one residue anchor, which would make this enzyme—*E. coli* gyrase—an outlier. 

Moreover, the water–metal ion bridge appears to play a positioning, rather than a binding, role because high concentrations of the quinolone were unable to induce high levels of DNA cleavage in the presence of the mutant enzymes and the quinolone was able to compete out the quinazolinedione and lower cleavage seen when both drugs were present as compared to when only the quinazolinedione was present. A positioning role for the water–metal ion bridge in *E. coli* gyrase is consistent with the function of the bridge in *E. coli* topoisomerase IV [29]. In contrast, previous work with *B. anthracis* topoisomerase IV [28,30] and gyrase [32] found the water–metal ion bridge to function as a binding interaction in that Gram-positive species. In addition, in *M. tuberculosis*, an acid-fast bacterium that contains only gyrase, the water–metal ion bridge was also found to play a binding role [31]. Taken together, these findings raise the possibility that the water–metal ion bridge has a split of function down Gram classification lines, with it functioning primarily as a binding interaction in Gram-positive type II topoisomerases and primarily as a positioning interaction in Gram-negative type II topoisomerases. We are currently working to test for the presence and the function of the water–metal ion bridge in other common pathogenic Gram-positive and Gram-negative bacterial species to determine whether the water–metal ion bridge has the same function in both type II topoisomerases from a given species, and more importantly, whether the function of the bridge in a given species can be predicted based on Gram classification. If there is indeed a split of function along Gram classification lines, then it may be necessary to approach designing a quinolone-based drug that overcomes resistance to the most common mutations (i.e., those that facilitate formation of the bridge), from two different angles in order to solve the two different issues—binding and positioning.

## Data Availability

Data are contained within the article.
